# Intensity-modulated radiation therapy using static ports of tomotherapy (TomoDirect): comparison with the TomoHelical mode

**DOI:** 10.1186/1748-717X-8-68

**Published:** 2013-03-21

**Authors:** Taro Murai, Yuta Shibamoto, Yoshihiko Manabe, Rumi Murata, Chikao Sugie, Akihiro Hayashi, Hiroya Ito, Yoshihito Miyoshi

**Affiliations:** 1 Department of Radiology, Nagoya City University Graduate School of Medical Sciences, 1 Kawasumi, Mizuho-cho, Mizuho-ku, Nagoya 467-8601, Japan; 2 Department of Radiation Oncology, JA Suzuka General Hospital, Suzuka, Japan

**Keywords:** Tomotherapy, TomoDirect, Intensity modulated radiotherapy, Lung cancer, Prostate cancer

## Abstract

**Purpose:**

With the new mode of Tomotherapy, irradiation can be delivered using static ports of the TomoDirect mode. The purpose of this study was to evaluate the characteristics of TomoDirect plans compared to conventional TomoHelical plans.

**Methods:**

TomoDirect and TomoHelical plans were compared in 46 patients with a prostate, thoracic wall or lung tumor. The mean target dose was used as the prescription dose. The minimum coverage dose of 95% of the target (D95%), conformity index (CI), uniformity index (UI), dose distribution in organs at risk and treatment time were evaluated. For TomoDirect, 2 to 5 static ports were used depending on the tumor location.

**Results:**

For the prostate target volume, TomoDirect plans could not reduce the rectal dose and required a longer treatment time than TomoHelical. For the thoracic wall target volume, the V5Gy of the lung or liver was lower in TomoDirect than in TomoHelical (*p* = 0.02). For the lung target volume, TomoDirect yielded higher CI (*p* = 0.009) but smaller V5Gy of the lung (*p* = 0.005) than TomoHelical. Treatment time did not differ significantly between the thoracic wall and lung plans.

**Conclusion:**

Prostate cancers should be treated with the TomoHelical mode. Considering the risk of low-dose radiation to the lung, the TomoDirect mode could be an option for thoracic wall and lung tumors.

## Introduction

The Tomotherapy Hi-Art system (Accuray Inc, Sunnyvale, CA, USA) is a radiation delivery system that combines dynamic intensity-modulated radiation therapy (IMRT) and an on-board imaging systems [1,2]. Treatment is usually delivered with 360-degree rotation of the 6-megavolt linear accelerator gantry. This conventional treatment mode is called TomoHelical. Recently, a new system upgrade named TomoDirect has been introduced [3,4]. TomoDirect allows the delivery of radiation at pre-established discrete angles with a fixed gantry. This new system is expected to reduce treatment time and reduce critical organ dose. However, these expectations have not yet been proven in clinical situations.

Clinically, localized prostate cancer is one of the most common malignancies treated with IMRT [5]. Using Tomotherapy, irradiation usually requires 3–6 minutes in addition to 10–15 minutes of set-up to treat prostate target volume, so only a limited number of patients can be treated with one Tomotherapy machine in one day. If TomoDirect could reduce the treatment time, more patients could be treated even in institutions possessing only one machine. In addition, Tomotherapy has recently been demonstrated to be adequate for treating moving targets with a hypofractionated course of radiotherapy [6-8]. In chemoradiotherapy for thoracic tumors using Tomotherapy, some reports about lung cancer [9] or mesothelioma [10-12] recommended that low dose exposure of the lung should be reduced as much as possible in order to reduce pulmonary toxicity. Thus, if TomoDirect could reduce the lung volume receiving low dose radiation, this modality could be expected to replace TomoHelical treatment in this situation.

In the present study, dose distributions and treatment times were compared between TomoDirect and TomoHelical plans in three clinical situations: a) prostate target volume, b) thoracic wall target volume and c) lung target volume. The purposes of this planning study were to examine 1) whether TomoDirect can reduce the treatment time and 2) whether TomoDirect can be an alternative to TomoHelical for prostatic and thoracic target volume.

## Methods

Comparisons of treatment plans between TomoDirect and TomoHelical were carried out during the fixed period from September 2010 to July 2011 at Suzuka General Hospital. All eligible patients seen during the period, i.e., 46 adults actually treated for prostate cancer, thoracic wall metastasis and lung tumor, were studied.

### CT Simulation

To reduce breathing motion and set-up error, patients were laid in the supine position on appropriate immobilization devices depending on the location of the radiotherapy target [13]. In planning computed tomography (CT), 2- to 3-mm slice thickness axial images were acquired using a 64-row multi-detector CT (Aquilion CX, Toshiba Medical, Otahara, Japan). Contrast-enhanced CT images were acquired and fused to the planning CT images to delineate the target, but unenhanced CT images were used for dose calculation to keep calculation accuracy [14]. Contouring of target volumes and normal structures was performed on the Pinnacle (3) version 9 treatment planning system (Philips Medical System, Eindhoven, Netherlands). The contours created in the treatment planning system were exported to the Tomotherapy Hi-Art treatment planning system v4.0, where TomoHelical and TomoDirect plans were generated.

### Planning

The prescription dose was defined as the mean dose of the planning target volume (PTV) (Dmean). As dose constraints, 1) D95% > 90% of the prescribed dose and 2) V90% ≥ 95% were satisfied. D95% was defined as the minimum dose delivered to 95% of the PTV. V90% was defined as the percentage of the PTV receiving at least 90% of the prescribed dose. Appropriate dose constraints were implemented for inverse planning procedures. In the Tomotherapy planning system, the parameters set before optimization were field width, modulation factor and pitch. The same field width, pitch and modulation factor were used in both TomoHelical and TomoDirect plans. To reduce dose to the critical organs, for example the lung, blocking structures were added appropriately in the TomoHelical plan. All optimization procedures were carried out until breaking the PTV dose constraints or satisfying other organs dose constraints. When the PTV constraints were broken, the optimization was restarted all over again. All constraints were satisfied, or 4–5 processes of trials and errors were performed for each plan. A “fine” calculation grid (1.95 mm × 1.95 mm) was used for the final calculation process.

To compare TomoDirect and TomoHelical, a minimum dose of 95% of the PTV (D95%), the dose distribution in organs at risk and the treatment time were evaluated in the Tomotherapy planning system. Conformity index (CI) and uniformity index (UI) were calculated according to the following formulae [15-17].

(1) $$\mathrm{Uniformityindex}\left(\mathrm{UI}\right)=D5\%/D95\%$$

(2) $$\mathrm{Conformityindex}\left(\mathrm{CI}\right)=\left(V_{\mathrm{PTV}}/TV_{\mathrm{PV}}\right)/\left(TV_{\mathrm{PV}}/V_{\mathrm{TV}}\right)$$

In these formulae, abbreviations indicate as follows: *V*_*PTV*_ = PTV (cc), *TV*_*PV*_ = lesion volume (cc) covered by the prescribed isodose, *V*_*TV*_ = prescribed isodose volume (cc), *D5%* = minimum dose delivered to the 5% of the PTV. Lower CI indicates higher conformity, and lower UI indicates better homogeneity. An ideal CI and UI are both 1.

In the prostate plans, all patients had stage III prostate cancer according to the 7^th^ edition of TNM staging at clinical diagnosis [18]. Thus, the prostate and the seminal vesicle were contoured as the clinical target volume (CTV) according to our protocol [19]. The CTV was expanded by 6 to 8 mm for the PTV (7 mm in lateral direction, 8 mm in cranial-caudal and anterior directions and 6 mm in posterior direction). The prescribed dose was 74.8 Gy in 34 fractions. Five static ports (angles A: 36, 108, 180, 232 and 304 degrees) were used for the TomoDirect plan according to our protocol [19]. In addition, another TomoDirect plan using different angles (angles B: 0, 75, 135, 225 and 285 degrees) was generated for each patient to evaluate influences of port angles. As organs at risk, the rectum and the bladder were contoured on non-contrast enhanced CT. The rectum was contoured from 1 cm below to 1 cm above the PTV in the cranio-caudal direction. Dose constraints were: 1) rectum: V58.5 Gy < 18%, V38.5 Gy < 35%, maximum dose (Dmax) < 75.1 Gy and 2) bladder: V60 Gy < 20%, V40 Gy < 35%. The V Gy value represents the percentage volume receiving the specified dose, e.g., V60 Gy is the percentage volume receiving 60 Gy.

**Figure 1:**
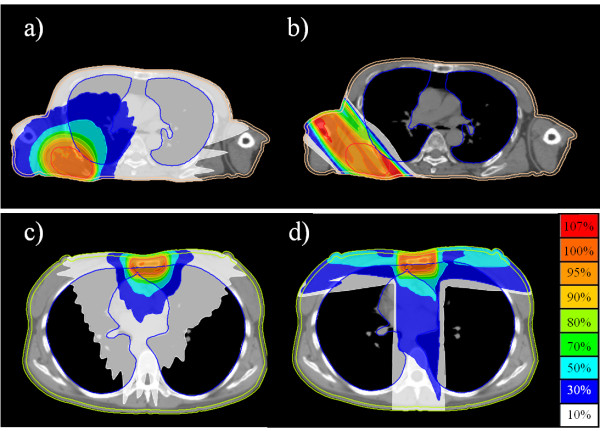
**Thoracic wall target volume.** Upper panel; the target volume located on the lateral wall was treated with TomoHelical mode (**a**) and TomoDirect mode (**b**). Lower panel; the target volume located near the midline was treated with TomoHelical mode (**c**) and TomoDirect mode (**d**).

In the thoracic wall plans, the visible enhanced lesion was contoured as the CTV. The CTV was expanded by 5 mm for the PTV and 39 Gy in 13 fractions was prescribed. For TomoDirect, oblique or 3 directions were used depending on the tumor location. Lesions located near the midline were treated with 3 ports of TomoDirect (Figure 1c, d). Organs at risk included the lung or liver, the skin and the spinal cord. The skin was contoured as a 3–5 mm thick layer under the body surface, and even when the PTV included the skin surface, it was spared to avoid skin toxicity. Spinal cord, lung and/or liver dose were reduced as much as possible.

In the lung plans, the visible enhanced lesion was contoured as the CTV. The CTV was expanded by 5 mm plus a patient-specific internal margin for the PTV. Each tumor motion was examined under 4-dimensional (4-D) CT during 2–3 respiratory cycles to make a maximum projection of the phases and delineate contours on that. Contrast material was not used at 4-D CT. To the PTV, 59.4 Gy in 27 fractions was prescribed. Four static ports were used for TomoDirect. Organs at risk included the lung, the skin and the spinal cord. Dose constraints were: 1) lung: mean lung dose (MLD) < 17 Gy, V10 Gy < 40%, V20 Gy < 30% and 2) spinal cord + 5 mm margin: Dmax < 50 Gy.

Comparisons of dose-volume parameters and treatment time between TomoDirect and TomoHelical plans were carried out using the two-tailed paired t-test. We assumed that the study populations distributed normally. All statistical analyses were performed using Statview 5.0 (SAS Institute Inc, Cary, NC). Statistical significance was defined as *p* ≤ 0.05. All planning and evaluation was performed by one radiation oncologist (T. M.). All doses evaluated in this study were calculated physical doses on the planning workstation.

## Results

**Figure 2:**
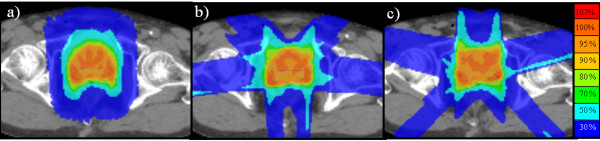
**Prostate target volume.** (**a**) TomoHelical plan. (**b**) TomoDirect plan – Angles A. (**c**) TomoDirect plan – Angles B.

**Figure 3:**
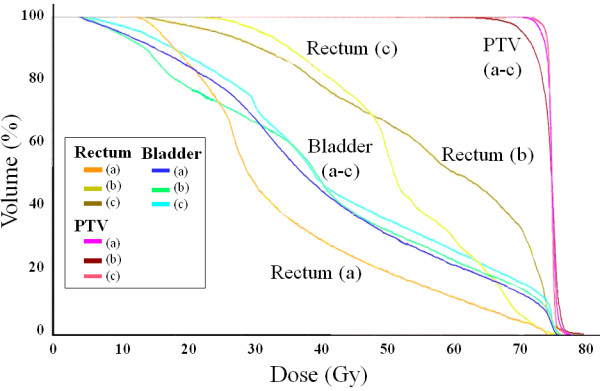
**Dose volume histograms of TomoHelical and TomoDirect plans for prostate target volume.** (**a**) TomoHelical plan. (**b**) TomoDirect plan – Angles A. (**c**) TomoDirect plan – Angles B.

A typical dose distribution and a typical dose volume histogram of the prostate plans are shown in Table 1, Figure 2 and Figure 3. Table 1 summarizes the treatment parameters, dose-volume parameters and treatment times of the two plans in 19 patients. D95%, CI and UI were almost equal. The dose distributions of the bladder were similar between TomoDirect (angles A) and TomoHelical plans; only V60 Gy and V70 Gy of the bladder in the TomoDirect plan using angles B were significantly higher than those in the TomoHelical plan. On the other hand, the V30, 40, 50, 60 and 70 Gy of the rectum in both of the TomoDirect plans were significantly higher than those in TomoHelical. Irrespective of the angles, TomoDirect plans could not satisfy the initial dose constraints. In TomoHelical plans, the rectal dose exceeded the prescribed dose (74.8 Gy) in 16 of 19 patients. In TomoDirect plans, the rectal dose exceeded 74.8 Gy in 14 patients for angles A and 17 patients for angles B. However, the rectal volumes receiving > 74.8 Gy were less than 1 cc in any of the cases. Beam-on times in both TomoDirect plans were longer than those in TomoHelical. No significant differences between these two TomoDirect plans were observed in the dose distribution of the bladder and rectum, CI, UI and beam-on time.

**Table 1:** Prostate target volume

		**TomoHelical**	**TomoDirect**			
			**Angles A ( *p*-value )**		**Angles B ( *p*-value )**	
		**(Mean ± standard deviation)**				
Patient number		19				
PTV^*^ (cc)		95.4 ± 22				
Modulation factor		2				
Pitch		0.215				
Field width		2.5				
Conformity index		1.95 ± 0.27	2.08 ± 0.37	(0.22)	2.01 ± 0.5	( 0.61)
Uniformity index		1.06 ± 0.04	1.07 ± 0.05	(0.26)	1.06 ± 0.04	(0.4)
D95% (%)		96 ± 3	96 ± 3	(0.99)	96 ± 2	(0.64)
Time (sec)		202 ± 15	221 ± 19	*(****0.001****)*	215 ± 23	***(0.04)***
Rectum	V10 Gy (%)	99 ± 2	99 ± 2	(0.51)	98 ± 3	(0.32)
	V20 Gy (%)	78 ± 15	87 ± 11	***(0.035)***	81 ± 12	*(0.49)*
	V30 Gy (%)	50 ± 4	65 ± 22	***(0.005)***	60 ± 19	***(0.03)***
	V40 Gy (%)	30 ± 4	46 ± 24	***(0.008)***	42 ± 21	***(0.01)***
	V50 Gy (%)	21 ± 3	33 ± 18	***(0.008)***	34 ± 19	***(0.01)***
	V60 Gy (%)	15 ± 2	22 ± 12	***(0.006)***	25 ± 16	***(0.01)***
	V70 Gy (%)	7 ± 2	10 ± 5	***(0.01)***	14 ± 12	***(0.02)***
Bladder	V10 Gy (%)	79 ± 18	76 ± 17	(0.62)	80 ± 17	(0.81)
	V20 Gy (%)	66 ± 18	61 ± 16	(0.39)	63 ± 19	(0.76)
	V30 Gy (%)	51 ± 14	50 ± 13	(0.89)	51 ± 14	(0.81)
	V40 Gy (%)	35 ± 9	37 ± 10	(0.5)	38 ± 9	(0.44)
	V50 Gy (%)	24 ± 6	27 ± 8	(0.23)	28 ± 7	(0.07)
	V60 Gy (%)	16 ± 4	19 ± 5	(0.1)	20 ± 5	***(0.01)***
	V70 Gy (%)	9 ± 2	10 ± 3	(0.28)	12 ± 3	***(0.004)***

^*^ PTV, planning target volume.

Each TomoDirect plan (angles A and angles B) was compared with TomoHelical plan.

In the thoracic wall plans (Figure 1), although the CI in TomoDirect plans was worse than that in TomoHelical (*p* = 0.004), D95% in TomoDirect was better than that in TomoHelical (97 ± 1% vs. 96 ± 1%, *p* = 0.04). The V5 Gy of the lung or liver in TomoDirect was lower than that in TomoHelical (19 ± 6% vs. 44 ± 8%, *p* = 0.02) (Table 2).

**Table 2:** Thoracic wall target volume

		**TomoHelical**	**TomoDirect**	***p-*value**
		**(Mean ± standard deviation)**		
Patient number		9 (Tangential: 6, 3 ports: 3)		
PTV^*^ (cc)		365 ± 319		
Modulation factor		1.8 - 2.0		
Pitch		0.25 - 0.287		
Field width		2.5 - 5.02		
Conformity index		2.21 ± 0.14	4.63 ± 0.61	*0.004*
Uniformity index		1.07 ± 0.04	1.06 ± 0.02	0.93
D95%		96 ± 1	97 ± 1	*0.04*
Time (sec)		259 ± 39	271 ± 34	0.72
Lung	MLD^†^(Gy)	6 ± 1	3 ± 1	*0.05*
	V5 Gy (%)	44 ± 8	19 ± 6	*0.02*
	V10 Gy (%)	22 ± 5	12 ± 4	0.38
	V20 Gy (%)	6 ± 1	6 ± 2	0.85
	V30 Gy (%)	2 ± 1	3 ± 1	0.65
Cord (maximum dose)		12 ± 3	7 ± 8	0.11

^*^PTV, planning target volume.

^†^MLD, mean lung dose.

**Figure 4:**
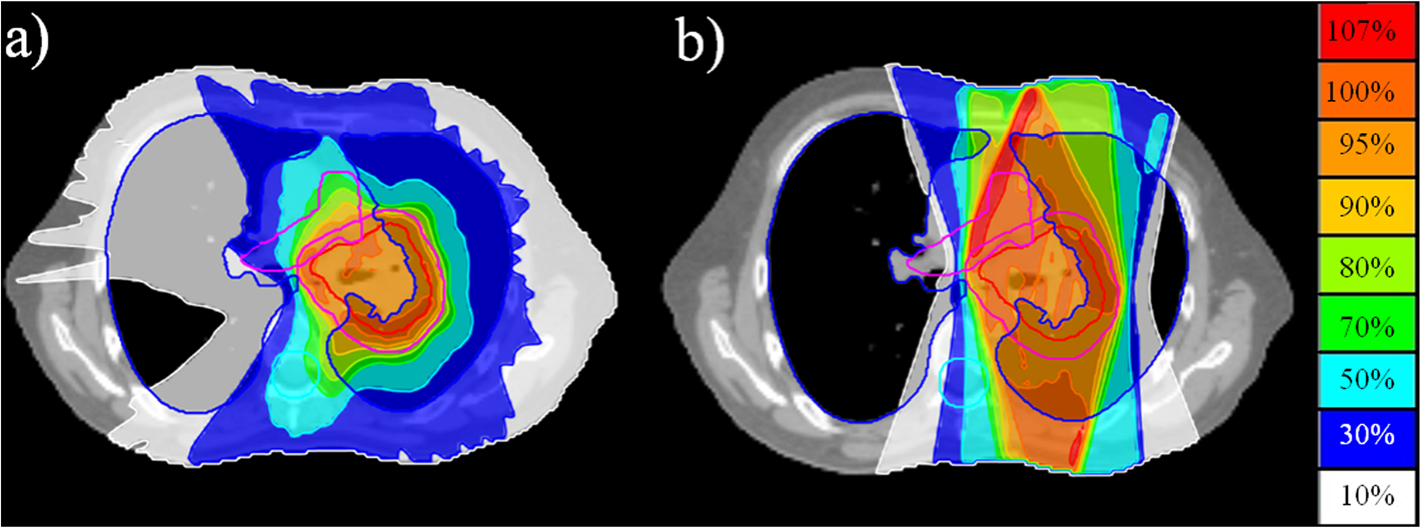
**Lung target volume.** (**a**) TomoHelical plan. (**b**) TomoDirect plan.

In the lung plans (Figure 4), D95%, UI and MLD did not differ significantly between the two modes, but the CI in TomoDirect was inferior to that in TomoHelical (3.24 ± 0.30 vs. 2.33 ± 0.13, *p* = 0.009). The V5 Gy of the lung was smaller than that in TomoHelical (30 ± 3% vs. 43 ± 3%, *p* = 0.005). Beam-on time did not differ significantly between TomoDirect and TomoHelical in thoracic wall and lung plans (Table 3).

**Table 3:** Lung target volume

		**Tomohelical**	**Tomodirect**	***p*-value**
		**(Mean ± standard deviation)**		
Patient number		18		
PTV^*^ (cc)		289 ± 327		
Modulation factor		1.8 - 2.2		
Pitch		0.172 - 0.43		
Field width		2.5 - 5.02		
Conformity index		2.33 ± 0.13	3.24 ± 0.30	*0.009*
Uniformity index		1.08 ± 0.01	1.06 ± 0 01	0.61
D95% (%)		95± 1	96 ± 1	0.23
Time (sec)		217 ± 16	235 ± 21	0.3
Lung	MLD^†^ (Gy)	10 ± 1	11 ± 1	0.68
	V5 Gy (%)	43 ± 3	30 ± 3	*0.005*
	V10 Gy (%)	29 ± 2	23 ± 2	0.1
	V20 Gy (%)	16 ± 2	18 ± 2	0.55
	V30 Gy (%)	10 ± 1	13 ± 2	0.15
	V40 Gy (%)	7 ± 1	8 ± 1	0.25
	V50 Gy (%)	4 ± 1	6 ± 1	0.14
Cord (maximum dose)		36 ± 3	38 ± 3	0.33

^*^PTV, planning target volume.

^†^MLD, mean lung dose.

## Discussion

The present study clarified three characteristics of the TomoDirect mode. First, in most cases, TomoDirect cannot reduce total treatment time. TomoDirect uses static gantry positions combined with simultaneous couch translation and multileaf collimator modulation. After a patient is treated from one gantry angle, the gantry is rotated to a different beam direction and the patient again passes through the bore for delivery of the subsequent port [3]. As a result, it takes more time to deliver the beams as the number of ports increases. Even 2- to 4-port TomoDirect plans needed almost equal beam-on time to TomoHelical plans. Thus, when 5 or more ports are used in TomoDirect plans, the beam-on time may often exceed that of TomoHelical.

Secondly, prostate target volume should be treated with TomoHelical. Davidson et al. [20] compared TomoHelical and 7-static-port conventional IMRT plans for prostate target volume. In their report, conventional IMRT plans were similar to TomoHelical plans. In contrast, in the current study, the dose to the rectum in TomoDirect plans was significantly higher than those in TomoHelical plans (Table 2, Figure 3). These results can be explained by a mechanical feature of Tomotherapy, which uses 64-leaf binary multileaf collimators. One leaf of the multileaf collimators is considered to have 51 beamlets associated with it during each gantry rotation [1,2]. Meanwhile, in TomoDirect plans, treatment delivery is limited to fewer directions with a smaller set of beamlets [3,4]. Therefore, it was possible that 5-static-port TomoDirect plans had fewer beamlets than 7-static-port conventional IMRT plans, resulting in TomoDirect mode being unable to reduce dose to the rectum in most cases with stage III prostate cancer in this study. Port angles might not have striking effects on the rectal dose in these situations. Furthermore, the treatment time in TomoDirect plans was longer than that in TomoHelical plans (Table 1). These results suggest that TomoHelical should be used for prostate target volume.

Third, considering the risk of low-dose radiation to the lung, TomoDirect mode is one option for thoracic wall and lung target volumes. In the current study, the TomoHelical plans delivered low-dose radiation to larger lung volumes than the TomoDirect plans. It was previously reported that, in comparison with 3D conformal radiotherapy, the use of TomoHelical plans resulted in delivery of low dose to areas in the body that would normally receive only scatter dose [4,21,22]. These results indicate the possibility that TomoDirect mode can reduce the lung toxicity.

Combined chemoradiotherapy is becoming a standard of care for the non-operative management of a variety of solid malignancies [23,24]. However, modeling tools to analyze the possible interactions between these modalities are lacking. Vogelius et al. [25] indicated that chemotherapy might increase the lung toxicity of low dose radiation exposure when a certain level of chemotherapy-related normal tissue damage is exceeded. As an example of this, among the first 46 patients treated with TomoHelical in a single-arm phase I/II dose-per-fraction escalation trial, the incidences of grade 2 and 3 radiation pneumonitis were only 13% and 0%, respectively, despite the large-volume of disease treated with very high dose [21]. In contrast, Song et al. [9] reported 7 cases with grade 3 or greater radiation pneumonitis among 37 patients (19%) also treated with TomoHelical, but 24 of the 37 patients had received concurrent chemotherapy and 13 had received neoadjuvant chemotherapy, illustrating the impact of chemotherapy. Thus, in cases undergoing chemoradiotherapy for a thoracic tumor, low-dose exposure of the lung, such as V5 Gy, should be reduced to the level of 3D conformal radiotherapy. According to the QUANTEC report [26], doses of the lung should be limited to the levels of V20 Gy < 30-35% and MLD < 20–23 Gy. In addition to these criteria, it is prudent to give attention to the low dose exposure of the lung.

In the current study, TomoDirect could also reduce the low-dose-exposed volume of the lung in treating thoracic wall and lung target volumes and achieve comparable target dose coverage. Thus, the TomoDirect mode may be an alternative in these situations. Further investigation into the clinical outcomes of these patients treated with TomoDirect mode is warranted.

## Conclusions

In conclusion, contrary to previous expectations, TomoDirect could not reduce treatment time in any of these three settings. Indeed, this modality should be used to reduce the critical organ dose. If chemotherapy is delivered, thoracic wall and lung target volumes may be a good indication for use of TomoDirect.

## Competing interests

The authors declare that they have no competing interests.

## Authors’ contributions

Each author had participated sufficiently in the work to take public responsibility for appropriate portions of the content. TM and YS designed the study. TM, YM, RM and HI collected the data. CS and AH interpreted the data and made some artworks. The manuscript was written by TM and YS; all other authors helped. All authors read and approved the final manuscript.
